# Analysis of mHealth research: mapping the relationship between mobile apps technology and healthcare during COVID-19 outbreak

**DOI:** 10.1186/s12992-022-00856-y

**Published:** 2022-06-28

**Authors:** Dina M. El-Sherif, Mohamed Abouzid

**Affiliations:** 1 National Institute of Oceanography and Fisheries (NIOF), Cairo, Egypt; 2 Department of Physical Pharmacy and Pharmacokinetics, Poznan University of Medical Sciences, 60-781 Poznan, Poland; 3 Doctoral School, Poznan University of Medical Sciences, 60-781 Poznan, Poland

**Keywords:** Healthcare, mHealth apps, Pandemic, Contact tracing, COVID-19, Bibliometric analysis

## Abstract

**Background:**

Mobile health applications (mHealth apps) offer enormous promise for illness monitoring and treatment to improve the provided medical care and promote health and wellbeing.

**Objective:**

We applied bibliometric quantitative analysis and network visualization to highlight research trends and areas of particular interest. We expect by summarizing the trends in mHealth app research, our work will serve as a roadmap for future investigations.

**Methods:**

Relevant English publications were extracted from the Scopus database. VOSviewer (version 1.6.17) was used to build coauthorship networks of authors, countries, and the co-occurrence networks of author keywords.

**Results:**

We analyzed 550 published articles on mHealth apps from 2020 to February 1, 2021. The yearly publications increased from 130 to 390 in 2021. *JMIR mHealth and uHealth* (33/550, 6.0%), *J. Med. Internet Res.* (27/550, 4.9%), *JMIR Res. Protoc.* (22/550, 4.0%) were the widest journals for these publications. The United States has the largest number of publications (143/550, 26.0%), and England ranks second (96/550, 17.5%). The top three productive authors were: Giansanti D., Samuel G., Lucivero F., and Zhang L. Frequent authors’ keywords have formed major 4 clusters representing the hot topics in the field: (1) artificial intelligence and telehealthcare; (2) digital contact tracing apps, privacy and security concerns; (3) mHealth apps and mental health; (4) mHealth apps in public health and health promotion.

**Conclusions:**

mHealth apps undergo current developments, and they remain hot topics in COVID-19. These findings might be useful in determining future perspectives to improve infectious disease control and present innovative solutions for healthcare.

## Introduction

mHealth is an acronym for mobile health, a term used to describe the use of mobile devices in the practise of medicine and public health to improve the quality of care and minimize costs to patients [1]. Smartphones are commonly used electronic products to identify consumer features, such as spatial and temporal trajectory and social contacts. In addition, they can be sued to deliver medical services through websites and applications (apps) like WeChat, Twitter, and Facebook, which was the beginning of ​​mobile health (mHealth) research. mHealth practices are primarily patient surveys, programs on chronic illness, and health promotion in conjunction with infectious diseases [2]. This technology can be applied to prevent and control infectious diseases; therefore, particular systems need to be built to leverage the critical knowledge needed, such as personal spatial-temporal trajectory data that can be obtained using Global Positioning System (GPS) and artificial geospatial intelligence (GeoAI) technologies [3].

mHealth provides innovative approaches to detect, monitor, and control infectious diseases and enhance the health system’s performance in tandem with internet-connected medical equipment [2]. The use of mHealth in the health sector has received considerable attention, especially during the outbreak of EVD, and may, in theory, lead to the implementation of the basic principles of Integrated Disease Surveillance and Response [4]. mHealth is committed to addressing many connectivity and management challenges and delays in communication and transport infrastructure-limited countries [5]. The World Health Organization study conducted in 2009 found that most Member States provide mobile communications to health contact centers and toll-free ambulance services, but these systems rarely use mHealth for monitoring, public awareness-raising, and decision support system. This needs increased capability and facilities and thus cannot be a financially limited health priority in affiliate States [6].

To evaluate cost-effectiveness, assessment is essential and requires educating the population about the benefits of mHealth, which relates to government policy. Despite the need for assessment, the survey showed that mHealth implementation results-based evaluation is not regularly done, and only 12% of Member States reported evaluation of mHealth services [7]. mHealth systems either use integrated or external sensors attached to the phone and establish a connected diagnostic device by their computational properties. The related diagnostic test identifies physical to molecular disease markers by sensors installed inside/outside the handset [2]. mHealth is now aggressively embracing mobile phones and smartwatches, with functional sensors to measure pulse, burned calories, and detect movement. Manufacturing companies introduce new sensors and imaging features to the latest wearable devices. Three-dimensional sensors, such as the infrared depth sensor device used in the iPhone X, can help in remote visual assessment [2, 8].

mHealth technologies could play a significant role in surveillance individuals infected with COVID-19, who are recommended to self-quarantine at home when they experience mild symptoms. mHealth technologies can detect poor prognosis and implement the appropriate therapeutic management before more problems arise. mHealth technologies are considered a powerful method to help in reducing or eliminating the spread of COVID-19 cases by using different monitoring tools.

In the current study we used bibliometric quantitative analysis and network visualization to highlight research trends, areas of particular interest, rising subjects, and cooperation partners in the field of mHealth apps. mHealth apps research is a new and exciting topic with a lot of promise for improving patient care and increasing health throughout the world. We hope that by summarizing the trends in mHealth apps research, our work will serve as a roadmap for future research initiatives, allowing us to progress this field of study even further.

## Methodology

### Search strategy and inclusion criteria

A literature search was performed on February 1, 2022, we used bibliometric quantitative analysis and network visualization to summarize the trends in mHealth app research. We applied the following query in the Scopus database:

(TITLE-ABS-KEY (“m*health” OR “mHealth” OR “mobile health app*” OR “tracing app*”) AND TITLE-ABS-KEY (COVID-19 OR SARS-CoV-2 OR nCoV-2 OR Coronavirus)).

Only publications published in journals were considered, and the language was limited to English. All of the data from the retrieved articles were combined in a CSV Excel file. The research strategy was validated by manually reviewing the retrieved papers to remove the duplicate publications and inspect data reliability [9].

### Bibliometric analysis

Bibliometric analysis often entails visualizing bibliometric maps [10]. It can identify hotspots in particular fields measure the productivity of authors, journals, countries, or institutions [11, 12]. Therefore, we deployed this method to visualize and analyze the bibliometric information in mHealth field. We calculated the frequency of co-occurrence of bibliographic information using co-word analysis. The clusters were performed using the co-occurrence information [10, 13]. Finally, the clusters were visualized graphically using VOSviewer version 1.6.17 (http://www.vosviewer.com/; Leiden University, Netherlands [14];) to build the bibliometric networks [10].

We constructed bibliometric maps that incorporated coauthorships of authors, countries/regions, and co-occurrence of author keywords using network mapping techniques. A circle with a label represents each node in a map – the larger the circle, the more frequent the item. The color of each circle (node) represents its cluster. The association strength between related nodes is represented by the length of edges (the line connecting two nodes).

### Research ethics

Scopus was used to search for and download data from bibliographic information. These were openly available data. No human participants or animal models were involved in the study. Therefore, no ethical approval was required.

## Results

### Source journals ranking

A total of 550 publications related to mHealth were retrieved from Scopus. The major theme was general health, focusing on medical informatics. Annual publications have been increased by almost 200% (from 130 to 390 in 2021). Two hundred ninety-six journals published these publications; Table 1 lists the top 10 contributing journals – accounting for 30.72% of all publications concerning mHealth during COVID-19. *JMIR mHealth and uHealth* published the most papers (33/550, 6.0%), followed by *J. Med. Internet Res.* (27/550, 4.9%), then *JMIR Res. Protoc.* (22/550, 4.0%).

**Table 1:** Top 10 journals publishing research on mHealth research, 2020-Feb. 1, 2022

Rank	CiteScore 2020	SNIP 2020	Journal	Country	Categories	Publications, n	Percentage^**a**^, %
1	6.3	1.705	JMIR mHealth and uHealth	Canada	Health Informatics	33	6.0
2	6.9	2.07	Journal of Medical Internet Research			27	4.9
3	2.3	0.469	JMIR Research Protocols			22	4.0
4	1.4	0.754	JMIR Formative Research			17	3.1
5	3.4	1.356	International Journal of Environmental Research and Public Health	Switzerland	Public Health, Environmental and Occupational Health	15	2.7
6	5.3	1.349	PLoS ONE	United States	Multidisciplinary Science	15	2.7
7	4.8	1.421	IEEE Access		Computer Sciences	13	2.4
8	6.2	1.630	JMIR Public Health and Surveillance	Canada	Health Informatics	10	1.8
9	14.9	3.116	IEEE Internet of Things Journal	United States	Computer Sciences	9	1.6
10	7.1	1.377	Scientific Reports	United Kingdom	Multidisciplinary Science	8	1.5

^a^The total number of retrieved papers on mHealth from 2020 to Feb. 1, 2022 (*N* = 550) was used as the denominator

### Coauthorship of countries

**Fig. 1:**
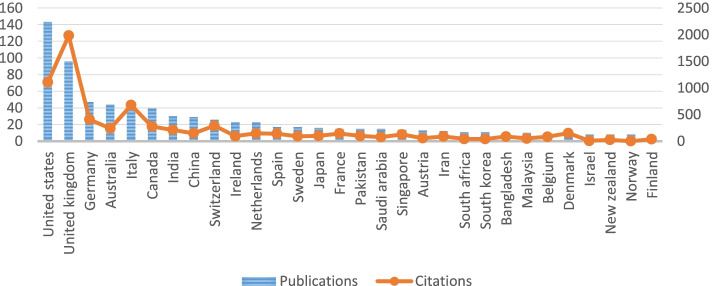
Top 30 countries/regions publishing mHealth research, 2020-Feb. 1, 2022

**Fig. 2:**
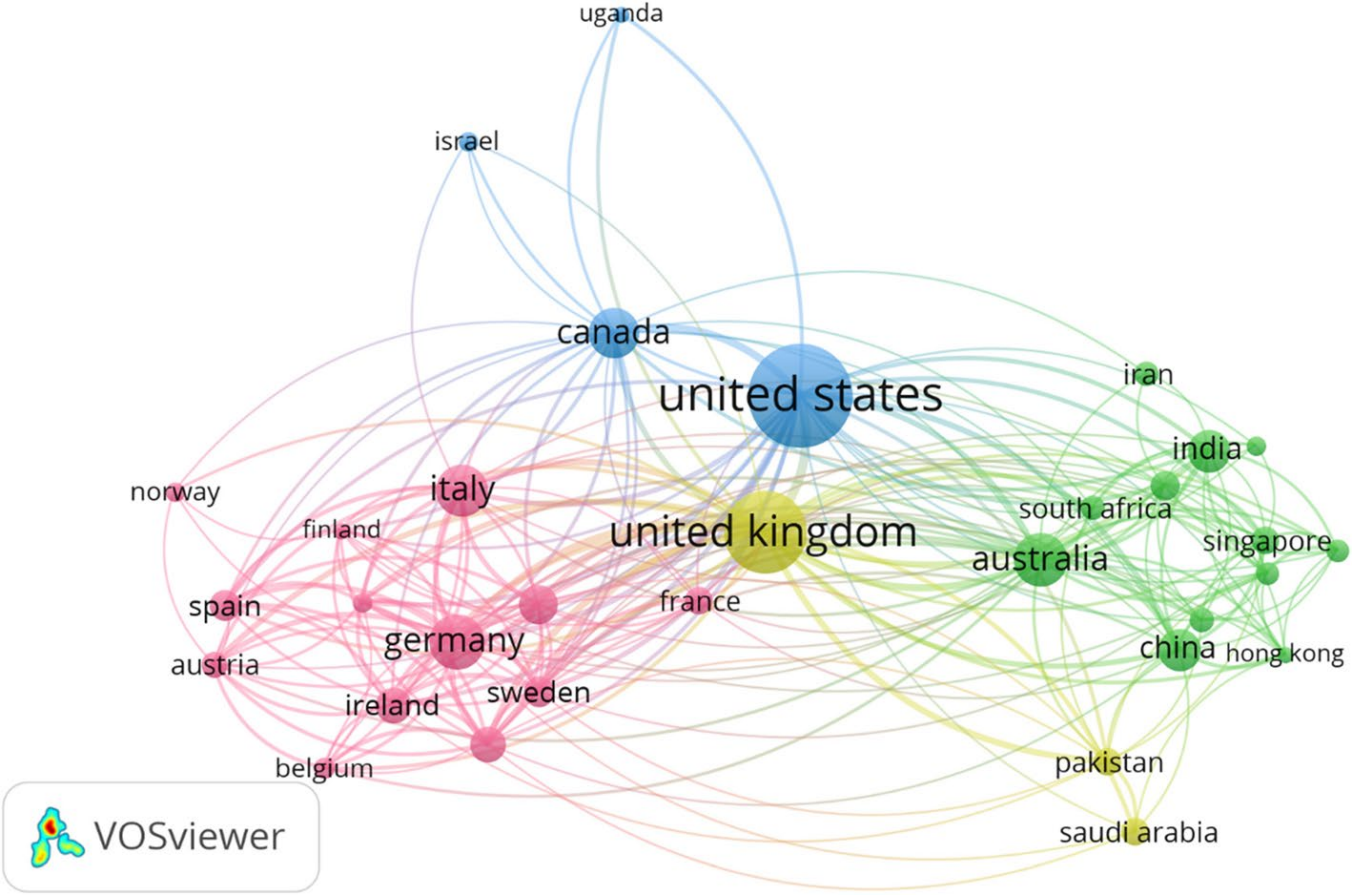
The coauthorship network of countries/regions that contributed to mHealth research, 2020-Feb. 1, 2022

We identified 74 countries contributing to retrieved publications. Figure 1 shows the top 30 countries publishing mHealth research and their citations. The United States has the most significant number of publications (143/550, 26.0%) and England ranks second (96/550, 17.5%), followed by Germany (47/550, 8.5%) and Australia (44/550, 8.0%). The collaboration between the highest 32 productive countries is visualized in Fig. 2, representing four distinct clusters.

### Coauthorship of authors

**Fig. 3:**
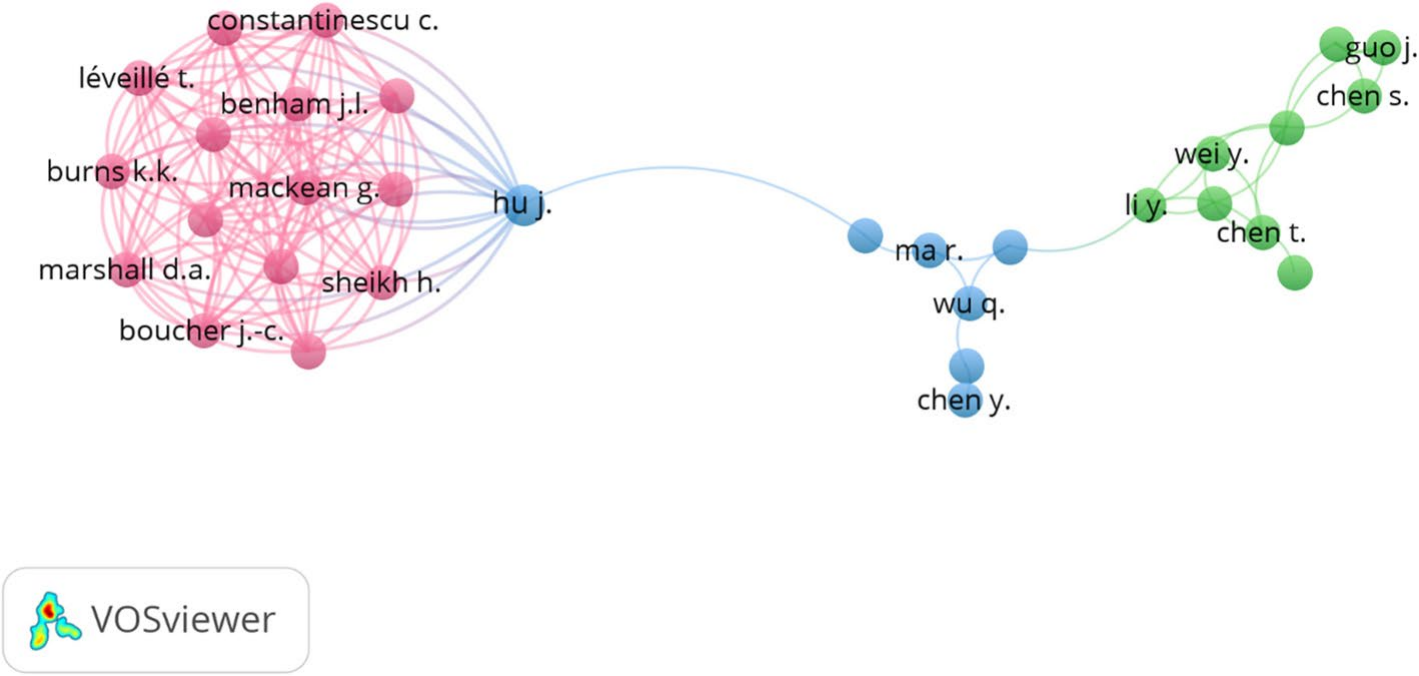
The coauthorship network of authors who contributed to mHealth research, 2020-Feb. 1, 2022

Overall, 2770 authors wrote 550 publications – only 202 authors had published at least two papers. The top 10 authors are listed in Table 2. The first authors were Giansanti D. and Samuel G. (6 publications), followed by Lucivero F. (5 publications), Zhang L. (4 publications), and Aguilera A. (3 publications). Further analysis shows the most extensive coauthorship network was 31 authors visualized in 3 clusters Fig. 3.

**Table 2:** Top 10 most productive authors in mHealth research, 2020-Feb. 1, 2022

Rank	Author	Publications, n	Citation, n
1	Giansanti D.	6	19
2	Samuel G.	6	23
3	Lucivero F.	5	23
4	Zhang L.	4	25
5	Aguilera A.	3	58
6	Ahmed R.M.	3	12
7	Ahsan N.	3	12
8	Chen X.	3	46
9	Choi S.W.	3	6
10	Figueroa C.A.	3	58

### The areas of particular interest

**Fig. 4:**
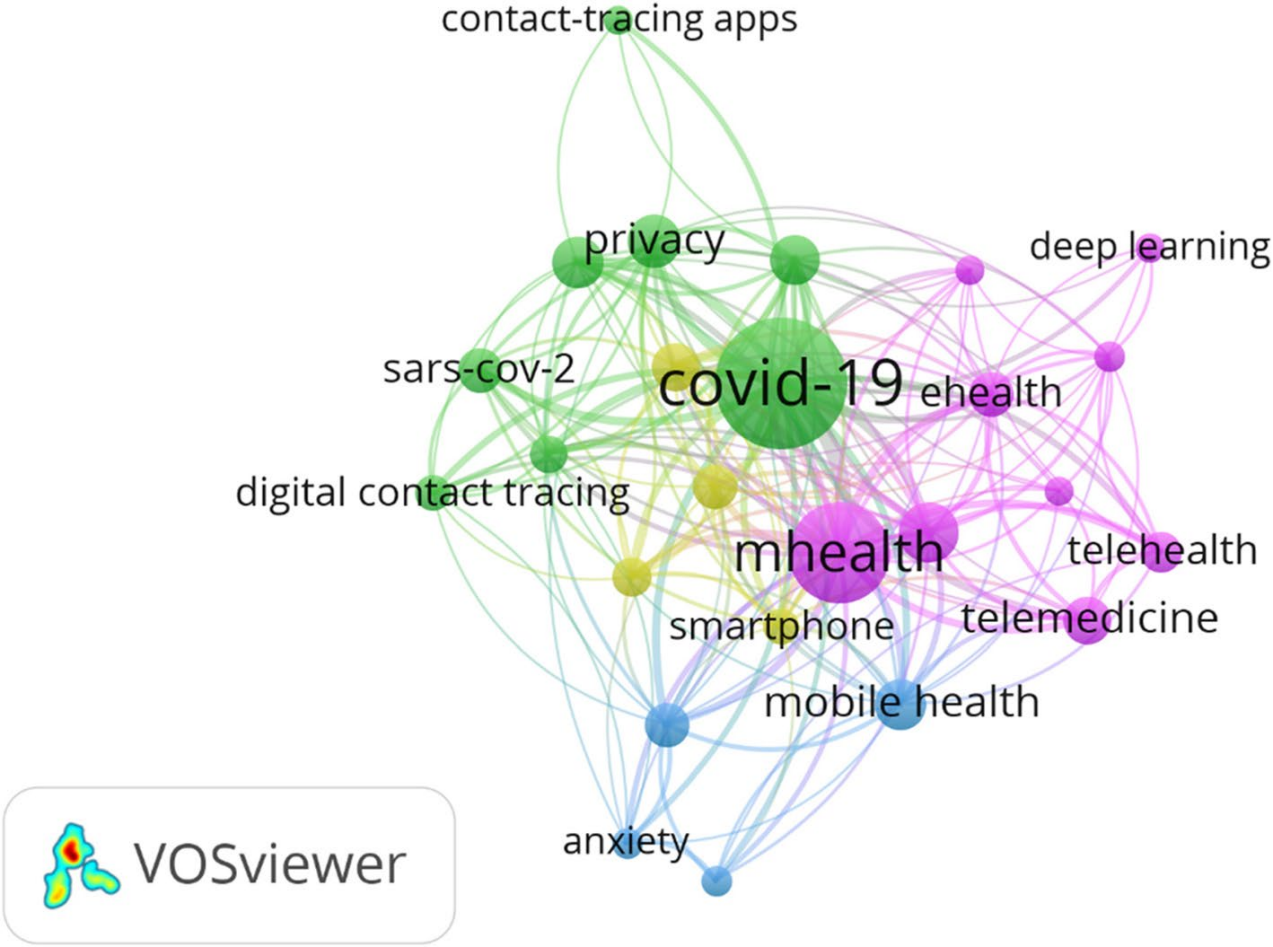
The co-occurrence network of author keywords in mHealth research, 2020-Feb. 1, 2022

We used the author keywords that were mentioned at least ten times to identify the areas of particular interest in the field. The highest frequently used keywords are COVID-19 (302), mHealth (165), digital health (50), and SARS-CoV-2 (27). The colors represent the 4 clusters: purple (cluster 1), green (cluster 2), blue (cluster 3), and yellow (cluster 4) as shown in Fig. 4.

## Discussion

### mHealth apps linked coauthorship networks

The visualized coauthorship maps highlight the collaboration status between researchers and countries. They also shape the current opportunities and possibilities for future collaboration.

The United States was the most significant country published in mHealth research field. The United States, United Kingdom, Germany and Australia were the current global leaders in mhealth app research as they published around 60.0% (330/550) of research concerning mHealth. Figure 2 reflects the extended collaboration with other Europe countries such as Germany, Austria, Spain, France and Italy (dominant of the purple cluster), and the Asian countries such as China, India, Iran and Singapore (dominant of the green cluster). The distribution of clusters also explains the regional properties of collaboration. European institutions tend to work together, similar to Asian institutions.

The initial high number of authors contributing to papers was 2770 authors. However, the best-visualized network highlighted only the collaboration between 31 authors with three clusters (Fig. 3). Therefore, mHealth field is attractive to many authors while there is a lack of collaboration. Those who dominate the purple cluster perform extensive research together. They are collaborating with only one co-author from the blue cluster. Another explanation could be that the field is highly specialized, and only experts or researchers within one research group or institution can perform their work together.

### The areas in particular interest in mHealth apps research

Keywords co-occurrence frequency analysis provides information about the broad topics in each field. However, having new frequent keywords in a specific timeline indicate the trending topics [15]. As shown in Fig. 4, four clusters of mHealth app articles are highlighted and discussed in the following sections

#### Artificial intelligence and telehealthcare

Cluster 1 (purple nodes) primarily focuses on AI and telehealthcare, including 9 high-frequency keywords, such as AI, deep learning, machine learning (ML), digital health, telemedicine, telehealth, and eHealth. Currently, smartphones are rapidly utilizing the potential of AI and ML to offer reliable and real-time insight into diverse areas of healthcare. Smartphones can use GPS or Bluetooth technology for touch monitoring purposes. While the practical implementation of contact tracing can seem challenging, previous epidemics have been successfully regulated by contact tracing and isolation initiatives [16].

The Chinese National Health Commission and China Electronics Technology Group Company are releasing the Close Contact Detector app, which uses big data from public authorities to track people during transportation and traveling; in addition to case reports, to verify if the user has had any close contact with confirmed or suspected cases in the recent past. The website will warn users about whether they worked together, shared the lecture room, stayed at the same building, or traveled by train or airplane with a person who was or has become suspect of the virus, based on their positioning and recent movement in their last 2 weeks (the presumed incubator period for COVID-19). A person can access ‘Close contact detector’ through three of China’s most common apps such as Alipay (payment app), WeChat (social media and mobile payment app), and QQ (social network app). The platform may pose some data protection concerns for some users, although it explicitly requests its customers to abide by China’s cybersecurity laws and not exploit private information [17, 18].

**Fig. 5:**
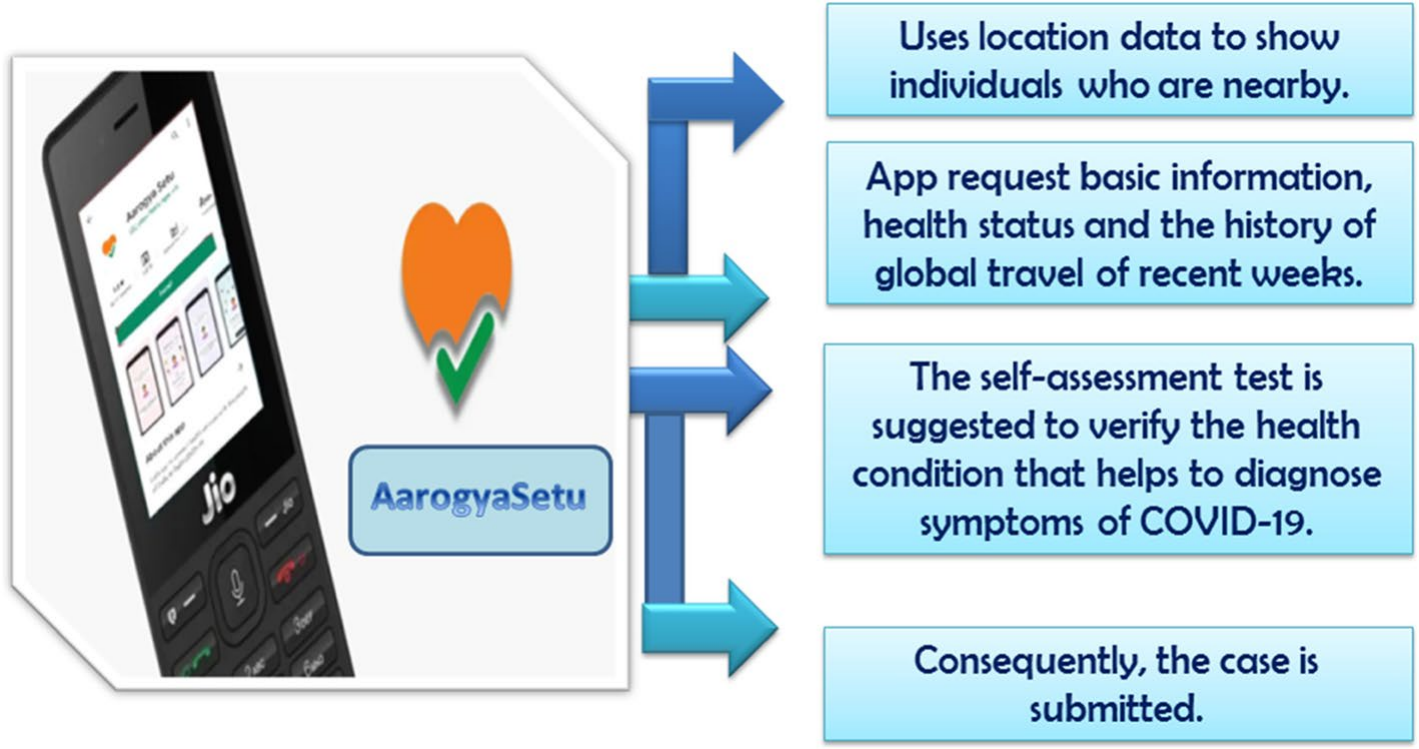
Working outline of the Indian AarogyaSetu App

The AarogyaSetu is surveillance software introduced by the Ministry of Electronics and IT in India to tackle COVID-19, enabling individuals to identify and determine their risk of coronavirus infection. It based on bluetooth technologies, ML algorithms, and AI. This software serves four main functions: it warns users when they are close to an infected human and reminds users of appropriate medical advice; it is highly secure and available in 11 languages on all standard operating systems, Android and iOS [19]. The outline of AarogyaSetu’s system is presented in Fig. 5. The officials contact the infected person and isolate him if it is necessary. If the case suffers from the symptoms but does not demand full quarantine, the person is recommended to isolate himself. If the individual is recovered, he/she shall be informed to continue practicing social distance. Furthermore, The AarogyaSetu app displays the person’s status all the time, offers an upgrade to COVID-19, and an E-pass feature allows an entity to apply for a passport to travel or move around during any lock-down. AqrogyaSetu is used to share best practices and frequently amended Advisories. All user inputs are registered in the National Informatics Center (NIC) secure servers along with their geographical location (via GPS) after obtaining the user’s proper permission to capture and analyze the data. An application programming interface can be provided by the server so that other apps can benefit from the shared data [20].

The nCOVnet is a deep neural network that can analyze lung X-ray images for fast detection of COVID-19 in X-Rays and detect whether a human is testing positive for the virus. In particular, the convolutional neural networks (CNN) have been tremendously influential in computer vision and medical image analysis tasks among different DL classifiers. CNN’s observations have proved its cogency in mapping image data to reliable and anticipated performance. As the primary focus of the virus is the lungs, analyzing the lungs’ modifications will provide a precise result of the existence of the virus [21]. Panwar et al. propose a model based on CNN capable of training photographs of corona virus-infected lungs and healthy lungs. By recognizing the symptoms of infected patients as hazy or shadowy spots in the X-ray images of the lungs, the proposed model can diagnose the COVID-19 cases at a faster level [21].

The proposed model can detect a positive COVID-19 patient in less than 5 seconds. With the small data they had, they could reach an actual positive rate of 97.62%. By adding more chest X-ray samples to the training data set, using the same model architecture already used, they can get higher precision. The unavailability of real-time RT-PCR kits is also resolved by nCOVnet, as it only includes an X-Ray system already available in most hospitals worldwide. Hence, countries will no longer wait for massive real-time RT-PCR kit shipments. Through the accelerated identification of COVID-19, we will contact and separate COVID-19 patients and eliminate population transmission [21].

The spatial distribution of diseases was identified and analyzed by GeoAI, and the impact of positional factors on disease outcomes was studied. For example, in an Ivory Coast sample of Africa, researchers tried to gather information from georeferenced mobile data on the prevalence and incidence factors of the Human Immunodeficiency Virus (HIV) through a machine. Such extracted characteristics are evaluated in relation to departmental HIV prevalence rates, where researchers have concluded that the predominant HIV conditions are correlated with variables, such as the phone usage region and total migration [22].

#### Digital contact tracing apps, privacy and security concerns

Cluster 2 (green cluster) focuses on digital contact tracing apps, privacy and security concerns, including eight high-frequency keywords such as contact tracing apps, SARS-CoV-2, coronavirus, pandemic, surveillance, and privacy.

Contact tracing is the method of identifying and recording people contaminated with COVID-19. Such approaches have resulted in inadequate detection of contacts and delays in communication tracing, such as identifying contacts involved in suspicious cases involving isolation [16]. Healthcare services can be provided across borders using technologies to facilitate communication between patients and physicians overseas. They can monitor, educate, diagnose, and even treat patients via video calls in a time- and cost-effective manner. Moreover, physicians can use these tools for collecting and storing data about their patients [23, 24].

Surveillance Outbreak Response Management and Analysis System (SORMAS) is a free Android and web-based App developed for handling laboratory samples and tests for case management, contact traceability, and surveillance [25]. Based on real-time health facility information, SORMAS helps surveillance officers and epidemiologists to diagnose diseases. SORMAS helps decision-makers, through public health officers, to promptly respond to take the appropriate action. Case and communication information is made freely available for intervention, quality assurance, and separation activities [26].

The SORMAS can be deployed and executed in regions that face the spreading of contagious diseases; also, the open-source design feature makes it possible to modify SORMAS taking into account particular countries’ public health requirements. SORMAS is seeking storage and processing of passive data for clinical trials in the future and monitoring of zoonotic diseases [27]. With the advent of COVID-19, Helmholtz Centre for Infection Research quickly established a virus identification and control module. In over 400 regions using SORMAS and countries’ entry points such as harbors and airports, the Ghana Health Service and Center for Disease Control of Nigeria instantly enabled this latest module to cover over 85 million people. For epidemiological surveillance and containment, the Nigerian Public Health Service uses SORMAS. SORMAS in Nepal is planning to be deployed for the COVID-19 pandemic. Fiji Island in other countries has disseminated SORMAS for COVID-19 [25].

TraceTogether is a smartphone App built by the Singaporean Ministry of Health (MOH) on March 20, 2020, to assist in COVID-19 contact tracking [28]. The software uses Bluetooth technologies to identify people who have been in direct contact with COVID-19 cases [29]. TraceTogether helps the MOH recognize who is logged close to confirmed cases of COVID-19; a contact tracer may investigate to identify suitable follow-up actions. Once TraceTogether sends a notification of a suspected contact, the Singapore MOH provides guidance, and the diagnosed person is not provided with more information. Thus, TraceTogether preserves each other’s identity except for what can be taken based on the entire contacts list of the user since the only data the user has is the binary display, which is the least possible data release for the device [30]. TraceTogether does not privilege consumers’ privacy from the authority but uses a comparatively high degree of confidence in the government for this purpose. It only collects the required data for contact tracing. For instance, GPS positioning information is not allowed to be used, and Bluetooth provides a good way to identify contacts. It does not attempt to conceal data from the Singaporean government. When a person has been infected and submits a list of close contacts to the MOH, the government may recall the telephone numbers of all persons with which the user has not been contacted. As a result, neither the diagnosed user nor the revealed contact has any anonymity whatsoever from the authorities [30]. There is no justification to distrust the TraceTogether team when they emphasize that people are not tracked, but their information could be used to do so. American citizens who believe in the officials are far less inclined to doubt than Singaporeans because the privacy trade-offs that Singaporeans can make will not be similar to what the Americans would consider [30].

The Australian Government launched the COVIDsafe App on April 26, 2020 to monitor communication and help health agencies quickly realize and resolve Coronavirus transmission [31]. The person installs the App to their mobile, and then the registration process requires them to provide their names (or pseudonym), personal information such as age, sex, phone number, and residential postal codes are then transferred from the users’ smartphone to the highly secure information storage system hosted in Australia. The data store transmits a PIN to the registrant when the registration information is received and accepted. The registrant then enters the PIN of the COVIDSafe App. After successful registration, an encrypted reference code is created for the COVIDSafe App on the consumer’s mobile phone. Every 2 hours, the encrypted reference code is changed. The final steps for the App are to allow Bluetooth to be activated on the mobile device and to ensure that “COVIDSafe operates while you are out or are possibly in touch with others” [32]. The Bluetooth feature of COVIDSafe ensures that a user’s smartphone sends a continuous stream of Bluetooth pings. A digital handshake happens when a user has activated smartphone pings another user’s enabled smartphone. This handshake is captured in encrypted form on every device the consumer has been in touch with. “The COVIDSafe website suggests that the proximity for direct contact is roughly 1.5 meters, for 15 minutes or more.” [32]. If a person is tested positive for COVID-19, he might be asked to use his uploaded data in the COVIDSafe database. It then proceeds a screening process and sends unencrypted contact tracing data to the applicable State or Territorial Health Authority, facilitating the contact tracing process [32].

Health care records, together with the financial record of an individual, patterns of social activity, and genomic information, are constantly being used, whether for legitimate businesses involved in direct advertising or individuals pursuing illegal access to services at the expense of others or for criminals who profit from the sale of identification bundled or invented. All the aforementioned information can be easily accessed globally via the internet from all types of computers, traditional desktops, cell phones, and wearables. Technology is going forward, but the obstacles are moving just as quickly. The ability to ensure confidentiality, integrity, transparency, and non-repudiation (identity authenticity) of the information offers unique advantages and risks. Cyber-attacks have become more complex, widespread, and successful as perimeter defenses have dropped. Standard authentication measures, such as efficient complicated passwords (when used), are insufficient for the modern wired environment [33].

Also, the number of digital health companies has grown, and many digital health apps and systems have become available at the industrial level; however, the quantity does not guarantee the quality. Therefore, DH companies should validate their digital health products and services by either healthcare providers or a hospital system to guarantee accuracy and efficiency. On top of that, privacy issues and limited access to DH could be the foremost challenges that digital health providers should address [34].

#### mHealth apps for mental health

The third cluster (blue) mainly concentrates on mHealth apps for mental health. It is composed of four major frequent words such as depression, mental health, anxiety, and mobile health. The novel coronavirus’s global spread has resulted in neuropsychiatric disorders such as suicidal deaths, depression, fear, anxiety, panic attacks, and a general decrease in overall wellbeing [35]. Similarly, people infected with COVID-19 are more likely to develop mental health problems because they face stigma and discrimination from their own family members [36]. mHealth apps can offer immediate assistance, confidentiality, personalization, and minimal cost. These qualities have the potential to increase access to mental health care and the equality of mental health resource allocation. mHeath apps may be used to offer online interventions for diagnosis, treatment, monitoring, and independent self-help mental health evaluation tools. Importantly, mHealth apps increase treatment accessibility by using momentary ecological assessment to remove obstacles to face-to-face help, particularly in patients suffering from depression, anxiety, stress, and other symptoms [37, 38].

#### mHealth apps in public health and health promotion

The fourth cluster (yellow) highlighted mHealth apps use in public health and health promotion with frequent mentioned keywords such as public health, smartphone, and mobile apps. mHealth applications may now give self-tracking capabilities due to the advancement of wearable technologies and smart sensors [39]. Wearable technology applications, such as weight management and physical activity tracking, are intended to help people prevent disease and enhance their overall well-being. People can keep track of weight, calories consumed, heart rate, respiration rate, exercise status, and how they feel or respond to therapy (e.g., side effects). Wearable devices are also used in hospital treatment and illness management. Some are used to improving medical care, mentioning patient recovery outside of the hospital to decrease the expenses. The wearable technology enables payers and provider organizations to boost and deliver real-time knowledge about medical services by expanding patients’ insights into providers. Wearable appliance demand might reach 27 billion dollars by 2022. This expansion creates enormous opportunities for disruption and innovation, both in the healthcare market and elsewhere [40]. People can utilize this type of tracking capabilities to encourage the adoption of healthy habits such as physical activity, a balanced diet, and obesity prevention [39, 41].

The public health sector has faced enormous challenges since the emergence of the coronavirus disease (COVID-19) pandemic in 2019. Digital health services have been widely adopted to respond to this public health emergency, which includes implementing comprehensive monitoring technologies, telehealth, creative diagnostic and therapeutic decision-making methods, greater penetration of wearable physiological parameters, and the development of broadcast networks to disseminate information related to COVID-19 to the public [12].

Precisely, mHealth technologies could predict the risk of infection and give preference to laboratory tests that indicate the probability of infection for each person [42]. Dester et al. assume that integrating three mHealth technologies (ePRO devices, smartwatches, and contact tracing apps) into an integrated, comprehensive mHealth approach could help in offering the ability to deploy an end-to-end solution that involves screening software, risk profiling, early warning, infection monitoring, tracking [42].

### The global perspective in the era of mHealth

mHealth, and the data generated as a result of it, when combined with analytics, provides a plethora of interesting potential for all stakeholders (patients, providers, and manufacturers). These prospects include but are not limited to, drug development facilitation and acceleration, diagnostic algorithm advancement, patient-reported data collecting, and a trend toward customized care. Furthermore, mHealth data may give real-world safety information, enhance predictive modeling, and aid comparative effectiveness studies, all of which can lead to considerable advantages for patients. Collaboration between healthcare providers, academic institutions, and the pharmaceutical sector, on the other hand, will be required to extend the usage of mHealth and the consequent influence the data may have on healthcare policy and delivery. There are several advantages for mHealth to meet unmet medical necessities, generate more patient-centric endpoints, and enhance existing trial endpoints; nevertheless, careful attention must be given to the inclusion of technology-derived metrics. As manufacturers explore using mHealth in pre or post-launch activities, it is necessary to create a digital health roadmap to guide the development, marketing, and implementation of mHealth to achieve optimum success.

### Strength and limitations

This research highlights new technologies, including detection, surveillance, and disease tracking, are powered by evidence and accessible at different stages of the COVID-19 pandemic. We also clarified the privacy and security issues that researchers need to know and examined the resources and strategies available to mitigate hazards. Concerning the limitations, the present data for analysis were only collected from Scopus (Elsevier), not from other search engines like WOS, PubMed, or Google Scholar (Google LLC). As a result, it’s conceivable that articles only available through one of these other search engines were overlooked. Only publications published in journals were considered, and the language was limited to English. The paper discusses the current advances in digital health services that enhance the management of infectious diseases; however, these technologies might be outdated in the future and will be replaced. Also, the research focuses only on trending mHealth apps during COVID-19 time. Other mHealth apps might be beneficial but were not trending during our analysis.

## Conclusion and recommendations

mHeath apps have played an important role during COVID-19 crisis. It helped countries to manage the spread of infection. There is a lack of collaboration between countries and authors worldwide, although mHealth field requires complex knowledge. Privacy issues remain a concern toward mHealth, and governmental and institutional regulations should provide a transparent policy toward using the users’ data.

Finally, awareness of digital health is required as a facilitator of medical care in many specialties, and the ability to rapidly change from in-person to virtual visits should help with chronic disease management and treatment continuity during infectious outbreaks and other significant disasters that interrupt traditional care models. We hope that this work will serve as a roadmap for future research paths to push this field of study further, and be a useful basis for identifying significant topics for future research, as well as potential for collaboration across research groups with comparable scientific interests in the field of the digital healthcare.

## Data Availability

Not Applicable.

## References

[1] Hoque MR, Rahman MS, Nipa NJ, Hasan MR (2020). Mobile health interventions in developing countries: a systematic review. Health Informatics J.

[2] Wood CS, Thomas MR, Budd J, Mashamba-Thompson TP, Herbst K, Pillay D (2019). Taking connected mobile-health diagnostics of infectious diseases to the field. Nature.

[3] Kamel Boulos MN, Peng G, Vopham T (2019). An overview of GeoAI applications in health and healthcare. Int J Health Geogr.

[4] Nasi G, Cucciniello M, Guerrazzi C (2015). The role of mobile technologies in health care processes: The case of cancer supportive care. J Med Internet Res.

[5] Fähnrich C, Denecke K, Adeoye OO, Benzler J, Claus H, Kirchner G (2015). Surveillance and outbreak response management system (SORMAS) to support the control of the Ebola virus disease outbreak in West Africa. Euro Surveill.

[6] Akhlaq A, McKinstry B, Bin MK, Sheikh A (2016). Barriers and facilitators to health information exchange in low- and middle-income country settings: a systematic review. Health Policy Plan.

[7] World Health Organization. New horizons for health through mobile technologies: second global survey on eHealth. Available from: https://www.who.int/goe/publications/goe_mhealth_web.pdf. [cited 2021 Feb 5].

[8] Chan PH, Wong CK, Poh YC, Pun L, Leung WWC, Wong YF (2016). Diagnostic performance of a smartphone-based Photoplethysmographic application for atrial fibrillation screening in a primary care setting. J Am Heart Assoc.

[9] Romero L, Portillo-Salido E (2019). Trends in sigma-1 receptor research: a 25-year bibliometric analysis. Front Pharmacol.

[10] van Eck NJ, Waltman L (2010). Software survey: VOSviewer, a computer program for bibliometric mapping. Scientometrics.

[11] Abouzid M, Główka AK, Karaźniewicz-Łada M (2021). Trend research of vitamin D receptor: bibliometric analysis.

[12] El-Sherif DM, Abouzid M, Elzarif MT, Ahmed AA, Albakri A, Alshehri MM (2022). Telehealth and Artificial Intelligence Insights into Healthcare during the COVID-19 Pandemic. Healthc.

[13] Agarwal A, Durairajanayagam D, Tatagari S, Esteves SC, Harlev A, Henkel R (2016). Bibliometrics: tracking research impact by selecting the appropriate metrics. Asian J Androl.

[14] van Eck NJ, Waltman L (2017). Citation-based clustering of publications using CitNetExplorer and VOSviewer. Scientometrics.

[15] Gan J, Cai Q, Galer P, Ma D, Chen X, Huang J (2019). Mapping the knowledge structure and trends of epilepsy genetics over the past decade: a co-word analysis based on medical subject headings terms. Medicine (United States).

[16] Danquah LO, Hasham N, MacFarlane M, Conteh FE, Momoh F, Tedesco AA (2019). Use of a mobile application for Ebola contact tracing and monitoring in northern Sierra Leone: a proof-of-concept study. BMC Infect Dis.

[17] China launches coronavirus “close contact detector” app - BBC News. Available from: https://www.bbc.com/news/technology-51439401. [cited 2021 Feb 17].

[18] Kamel Boulos MN, Geraghty EM (2020). Geographical tracking and mapping of coronavirus disease COVID-19/severe acute respiratory syndrome coronavirus 2 (SARS-CoV-2) epidemic and associated events around the world: how 21st century GIS technologies are supporting the global fight against outbreaks and epidemics. Int J Health Geogr.

[19] Abd-Alrazaq A, Alajlani M, Alhuwail D, Schneider J, Al-Kuwari S, Shah Z (2020). Artificial intelligence in the fight against COVID-19: Scoping review. J Med Internet Res.

[20] Jhunjhunwala A (2020). Role of telecom network to manage COVID-19 in India: Aarogya Setu. Trans Indian Natl Acad Eng.

[21] Panwar H, Gupta PK, Siddiqui MK, Morales-Menendez R, Singh V (2020). Application of deep learning for fast detection of COVID-19 in X-rays using nCOVnet. Chaos, Solitons Fractals.

[22] Brdar S, Gavrić K, Ćulibrk D, Crnojević V (2016). Unveiling spatial epidemiology of HIV with mobile phone data. Sci Rep.

[23] Nittari G, Khuman R, Baldoni S, Pallotta G, Battineni G, Sirignano A (2020). Telemedicine practice: review of the current ethical and legal challenges. Telemed e-Health.

[24] Senbekov M, Saliev T, Bukeyeva Z, Almabayeva A, Zhanaliyeva M, Aitenova N (2020). The recent progress and applications of digital technologies in healthcare: a review. Int J Telemed Appl.

[25] Tom-Aba D, Silenou BC, Doerrbecker J, Fourie C, Leitner C, Wahnschaffe M (2020). The surveillance outbreak response management and analysis system (SORMAS): Digital health global goods maturity assessment. JMIR Public Health Surveill.

[26] Tom-Aba D, Nguku PM, Arinze CC, Krause G (2018). Assessing the concepts and designs of 58 mobile apps for the management of the 2014-2015 West Africa Ebola outbreak: systematic review. JMIR Public Health Surveill.

[27] Yavlinsky A, Lule SA, Burns R, Zumla A, McHugh TD, Ntoumi F (2020). Mobile-based and open-source case detection and infectious disease outbreak management systems: a review. Wellcome Open Res.

[28] TraceTogether - behind the scenes look at its development process. Available from: https://www.tech.gov.sg/media/technews/tracetogether-behind-the-scenes-look-at-its-development-process. [cited 2021 Feb 15].

[29] Alwashmi MF (2020). The use of digital health in the detection and management of COVID-19. Int J Environ Res Public Health.

[30] Cho H, Ippolito D, Yu YW. Contact tracing mobile apps for COVID-19: privacy considerations and related trade-offs. arXiv. 2020; Available from: http://arxiv.org/abs/2003.11511. [cited 2021 Feb 15].

[31] Wang D, Liu F. Privacy risk and preservation For COVID-19 contact tracing apps. arXiv. 2020;33 Available from: http://arxiv.org/abs/2006.15433. [cited 2021 Feb 17].

[32] Watts D. COVIDSafe, Australia’s digital contact tracing app: the legal issues. SSRN Electron J. 2020; Available from: https://papers.ssrn.com/abstract=3591622. [cited 2021 Feb 26]. Elsevier BV.

[33] Filkins BL, Kim JY, Roberts B, Armstrong W, Miller MA, Hultner ML (2016). Privacy and security in the era of digital health: what should translational researchers know and do about it?. Am J Transl Res.

[34] Van Panhuis WG, Paul P, Emerson C, Grefenstette J, Wilder R, Herbst AJ (2014). A systematic review of barriers to data sharing in public health. BMC Public Health.

[35] Brooks SK, Webster RK, Smith LE, Woodland L, Wessely S, Greenberg N (2020). The psychological impact of quarantine and how to reduce it: rapid review of the evidence. Lancet.

[36] Poudel K, Subedi P (2020). Impact of COVID-19 pandemic on socioeconomic and mental health aspects in Nepal. Int J Soc Psychiatry.

[37] Donker T, Petrie K, Proudfoot J, Clarke J, Birch MR, Christensen H (2013). Smartphones for smarter delivery of mental health programs: a systematic review. J Med Internet Res.

[38] Lemey C, Larsen ME, Devylder J, Courtet P, Billot R, Lenca P (2019). Clinicians’ concerns about mobile ecological momentary assessment tools designed for emerging psychiatric problems: prospective acceptability assessment of the MEmind app. J Med Internet Res.

[39] Martin CK, Gilmore LA, Apolzan JW, Myers CA, Thomas DM, Redman LM (2016). Smartloss: a personalized mobile health intervention for weight management and health promotion. JMIR Mhealth Uhealth.

[40] Pawar MV, Pawar P, Pawar AM. HealthWare telemedicine technology (HWTT) evolution map for healthcare. Wearable Telemed Technol Healthc Ind. Academic Press. 2022. p. 17–32.

[41] Stavrositu C, Kim J (2018). self-persuasion through mobile applications: exploring different routes to health behavioral change. Cyberpsychol Behav Soc Netw.

[42] Adans-Dester CP, Bamberg S, Bertacchi FP, Caulfield B, Chappie K, Demarchi D (2020). Can mHealth technology help mitigate the effects of the COVID-19 pandemic?. IEEE Open J Eng Med Biol.

